# tRNA Modification Detection Using Graphene Nanopores: A Simulation Study

**DOI:** 10.3390/biom7030065

**Published:** 2017-08-25

**Authors:** Khadijah Onanuga, Thomas J. Begley, Alan A. Chen, Srivathsan V. Ranganathan

**Affiliations:** 1 SUNY Polytechnic Institute, Colleges of Nanoscale Science and Engineering, 257 Fuller Road, Albany, NY 12203, USA; konanuga-islam@sunypoly.edu (K.O.); tbegley@sunypoly.edu (T.J.B.); 2 Department of Biology, University at Albany, State University of New York, 1400 Washington Avenue, Albany, NY 12222, USA; 3 The RNA Institute, University at Albany, State University of New York, 1400 Washington Avenue, Albany, NY 12222, USA; achen6@albany.edu; 4 Department of Chemistry, University at Albany, State University of New York, 1400 Washington Avenue, Albany, NY 12222, USA

**Keywords:** tRNA modification, graphene, nanopore, wobble uridine

## Abstract

There are over 100 enzyme-catalyzed modifications on transfer RNA (tRNA) molecules. The levels and identity of wobble uridine (U) modifications are affected by environmental conditions and diseased states, making wobble U detection a potential biomarker for exposures and pathological conditions. The current detection of RNA modifications requires working with nucleosides in bulk samples. Nanopore detection technology uses a single-molecule approach that has the potential to detect tRNA modifications. To evaluate the feasibility of this approach, we have performed all-atom molecular dynamics (MD) simulation studies of a five-layered graphene nanopore by localizing canonical and modified uridine nucleosides. We found that in a 1 M KCl solution with applied positive and negative biases not exceeding 2 V, nanopores can distinguish U from 5-carbonylmethyluridine (cm^5^U), 5-methoxycarbonylmethyluridine (mcm^5^U), 5-methoxycarbonylmethyl-2-thiouridine (mcm^5^s^2^U), and 5-methoxycarbonylmethyl-2′-*O*-methyluridine (mcm^5^Um) based on changes in the resistance of the nanopore. Specifically, we observed that in nanopores with dimensions less than 3 nm diameter, a localized mcm^5^Um and mcm^5^U modifications could be clearly distinguished from the canonical uridine, while the other modifications showed a modest yet detectable decrease in their respective nanopore conductance. We have compared the results between nanopores of various sizes to aid in the design, optimization, and fabrication of graphene nanopores devices for tRNA modification detection.

## 1. Introduction

Chemical modifications are essential to the proper structure and function of RNA [1]. Ribosomal RNA (rRNA) and transfer RNA (tRNA) commonly undergo enzyme-catalyzed modifications to ensure the accuracy and efficiency of protein translation [2]. Over 109 RNA modifications to standard nucleosides are known, a few of which are acetylation, thiolation, alkylation, and base or ribose methylation [3]. tRNA modifications, in particular, play an important role in gene expression [1]. The tRNA molecule made of roughly eighty nucleosides is methylated at more than 20 of its nucleosides [2]. tRNA modifications in the wobble position 34 or position 37 that is 3′-adjacent to the anticodon nucleotides directly modulate codon recognition [1]. Also, the structure and chemistry of modified nucleosides facilitate the recognition of tRNAs by some aminoacyl-tRNA synthetases, making these modifications fundamental to tRNA folding, structural identity, and function [1,4]. 

**Figure 1:**
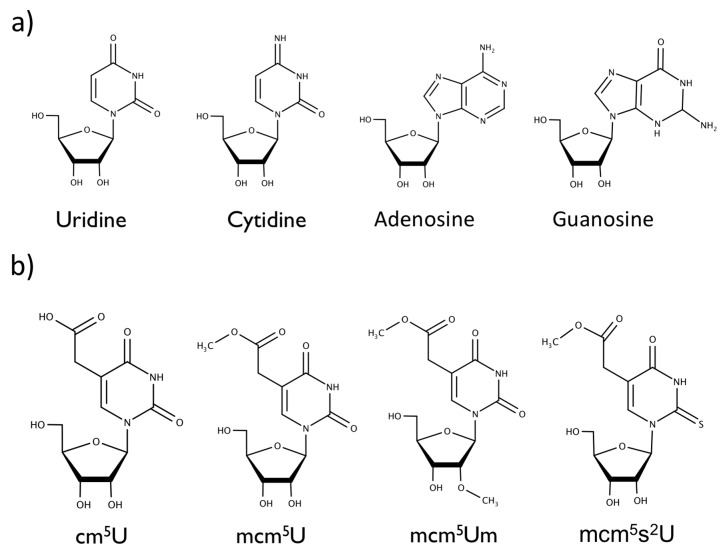
Chemical structures of the (**a**) canonical nucleosides uridine, cytidine, adenosine, guanosine, and (**b**) modifications to wobble uridine 34 in the anticodon stem loop, 5-carbonyl-methyluridine (cm^5^U), 5-methoxycarbonyl-methyluridine (mcm^5^U), 5-methoxycarbonyl-2′-*O*-methyluridine (mcm^5^Um), and 5-methylcarboxymethyl-2-thiouridine (mcm^5^s^2^U).

Translation modulated by tRNA modifications plays a crucial role during cellular stress responses [4,5]. Translation can be suppressed under certain physiological conditions like apoptosis, heat shock, oxygen shock (hypoxia or oxidative stress), DNA damage, nutrient deprivation, and some viral infections [6]. The cellular response is vital since reactive oxygen species (ROS) are known to contribute to cancers and neurodegenerative disorders [7,8,9,10]. In mammalian cells, selenoproteins are synthesized in direct response to oxidative stress [11]. Selenocysteine (SeCys), the main active component in selenoproteins, is a non-canonical amino acid that lacks its own codon for translation [12]. During selenoprotein synthesis, SeCys relies on the methylation of tRNA^[Ser]^ to tRNA^[Ser]Sec^ preventing the UGA stop from terminating translation, instead incorporating SeCys into the growing peptide chain [12]. tRNA^[Ser]Sec^ is unique and has four modified nucleosides, including pseudouridine (ψ), 1-methyladenosine (m^1^A), *N*^6^-isopentenyladenosine (𝑖^6^A), and 5-methylcarboxymethyl-uridine (mcm^5^U). The biochemical synthesis of the modified uridine (mcm^5^U) on tRNA^[Ser]Sec^ includes the intermediaries 5-methyluridine (m^5^U), 5-carbonylmethyluridine (cm^5^U), and mcm^5^U, all of which could be transiently found at wobble uridine position 34 of the anticodon stem loop (Figure 1) [12,13]. The mcm^5^U modification undergoes further methylation to 5-methoxycarbonylmethyl-2′-*O*-methyluridine (mcm^5^Um), which is only known to exist on the tRNA^[Ser]Sec^ [12]. Another closely related RNA modification is 5-methoxycarbonylmethyl-2-thiouridine (mcm^5^s^2^U), which is found on multiple tRNA species that decode codons for glutamic acid, lysine, and glutamine.

The levels and identity of wobble uridine (U) modifications are therefore indicative of the environmental conditions and diseased states [14,15,16], making wobble U detection a potentially useful biomarker for exposures and pathological conditions. The detection and quantification of tRNA modifications are vital for understanding disease pathways. Some of the earliest techniques used for separating and detecting modified nucleosides on tRNA are chromatography based [17]. Other techniques like high-performance liquid chromatography are sometimes used in tandem with mass spectrometry and stand-alone matrix-assisted laser desorption/ionization (MALDI) mass spectrometry [18]. Current chromatography-based techniques require multiple complex steps to isolate the nucleosides and can include the use of radioactively labeled components. Regardless, detection requires the separation of the modified components, which are then detected by mass and fragmentation patterns [17]. During the analytical steps, the original structure of the RNA is altered [17,19]. More techniques have emerged to eliminate the need to fragment RNA and avoid altering its original structure, which include antibody-based detection techniques. However, the affinity of antibodies to similar modifications in DNA or RNA result in cross reactivity, yielding ambiguous results [19,20]. Also, the immunogenicity of modified nucleosides is another drawback of antibody-based methods [19,20]. Alternative methods need to be developed to overcome the current disadvantages associated with the analysis of modified RNA nucleosides, namely having to fragment the RNA before modification detection, the semi quantitative nature of chromatography-based techniques, the length of time required, and the need for radioactive labeling [18]. 

The advent of nanotechnology has opened new frontiers in the application of solid-state nanopore sensors for the detection of RNA nucleosides. Nanopore sensors have the potential to achieve single molecule resolution in nucleobase and modification detection on RNA, as well as DNA, in a more accurate, faster, and more reproducible manner [21,22,23,24,25]. The idea of using nanopores for DNA sequencing was first proposed by Church, Deamer, Branton, Baldarelli, and Kasianowicz in 1995 and the application patented in 1998 [26]. Nanopore sensing technology has been shown to accurately capture single molecules measurements at microseconds temporal resolution. Nanopore based analysis of standard DNA nucleosides occurs with high sensitivity and high throughput, and has made it a rapidly developing field of research with applications in genome sequencing, drug discovery and development [27]. 

Nanopore technologies can include both biological and solid-state devices [28]. Biological nanopores made of proteins embedded in the cell membrane exist in all living systems, and they regulate the movement of molecules, ions, and electronic potential across the membrane [22,29,30]. Under an applied transmembrane voltage, an open protein channel can facilitate the selective transport of ions across the membrane generating electrical currents in the picoampere range that can be measured [22]. Biological nanopores have been used for DNA sequencing, identifying and characterizing solvated single molecules, and other capabilities as electronic sensors [29,30,31]. Some biological nanopores that have been extensively studied and modified for specific targeting include, heptameric protein α-Hemolysin (α-HL), octameric protein channel *Mycobacterium smegmatis* porin A (MspA) [26,30,32], and phi29 DNA packaging nanomotor protein in bacteriophage [33]. Proof-of-concept experiments by Smith et al. [34] show that linear tRNA attached to adapter phi29 DNA polymerase can translocate through a α-Hemolysin nanopore and give ionic current signals that are characteristic of the individual tRNA passing through the pore [34]. Researchers have reported using MspA to detect and map DNA methylation using 5-methylcytosine and 5-hydroxy-methylcytosine (hm^5^C) [35]. In addition, the connector protein of phi29 was modified, reconstituted into liposomes, and inserted into planar lipid bilayers for the translocation and detection of double-stranded DNA [33]. It is important to note that while some modified DNA nucleosides have been analyzed using nanopore technologies, the detection of modified RNA nucleosides has not been actively pursued, most likely due to the diversity of modifications and questions on sensitivity.

There are some notable challenges associated with biological nanopores. Functionalizing surfaces and controlling the geometry at the nanometer scale remain topmost on the list of challenges faced in the use of nanopore sensing for bioanalytical applications [36]. Biological nanopores like α-HL can be modified via genetic engineering to target specific nucleosides. However, the narrow diameter of the channel limits its application to the study of single-stranded nucleic acids and unfolded proteins. Challenges faced in the integration of biological nanopores into sensing devices include the mechanical instabilities of the lipid bilayers and controlling the amount of protein insertions into the lipid bilayers [36]. Unlike biological nanopores, solid-state nanopores are highly versatile sensing devices with superior thermal, mechanical, and chemical stability. Solid-state nanopores can be made from graphene, silicon nitride, silicon oxide, or aluminum oxide [26,37,38]. Solid-state nanopores are capable of faster, more accurate, and cheaper label-free detection and better analysis of single molecules compared to biological nanopores [38]. Graphene nanopores are unique with single-layer atomic thickness (~0.3 nm) and possess highly desirable qualities, such as mechanical stability and electrical properties, and hence are employed in a wide range of applications [34,39,40]. Larger blockage currents have been recorded in graphene nanopores than for traditional solid-state nanopores due to the unique atomic thinness of graphene [24]. The higher electrical conductivity of graphene also poses an advantage over the other solid-state nanopores, which tend to be rather insulating [24]. Graphene properties can be tuned by staking multiple layers to produce nanopores with varying thickness [24]. In nanopore sensing technology, graphene has been used as stacked nanolayers with nanopores of varying thickness to analyze nucleic acids sequences [21]. 

Combinations of experiments and simulations have revealed a wealth of knowledge on DNA translocation through nanopores [41]. Molecular dynamics simulations have been used to understand the role of nucleotide orientation and neighboring nucleotide chemistry on the passage of ions through the nanopore [42,43]. Drnjic and coworkers have shown that hm^5^C modification in a double stranded DNA can be distinguished from its canonical counterpart using a solid-state nanopore [44]. Aksimentiev and colleagues have studied translocation of 5-hydroxy-methyl-uridine containing DNA, and shown that the modification alters DNA flexibility and hydrophobicity, which in turn affect the translocation of the DNA molecule and the associated passage of ions through the nanopore [45]. Even though graphene-based nanopores are widely studied, to our knowledge, the feasibility of detection of modified RNA nucleosides using graphene nanopores has not been studied extensively. Our study employs molecular dynamics (MD) simulations that incorporate experimentally calibrated molecular models for RNA–graphene interactions to explore nanopore-based detection of modified nucleosides unique to tRNAs. We show that MD simulations can be used to predict changes in ionic current/pore resistance in response to localization of nucleosides in the nanopore environment. We studied a range of nanopore dimensions and modification chemistries and show the theoretical limit of detection of the nanopores. This study lays the foundation for more in-depth analyses of nanopore design and functionalization to optimize them for modified nucleoside detection and further guide their fabrication and characterization. 

## 2. Results and Discussion

**Figure 2:**
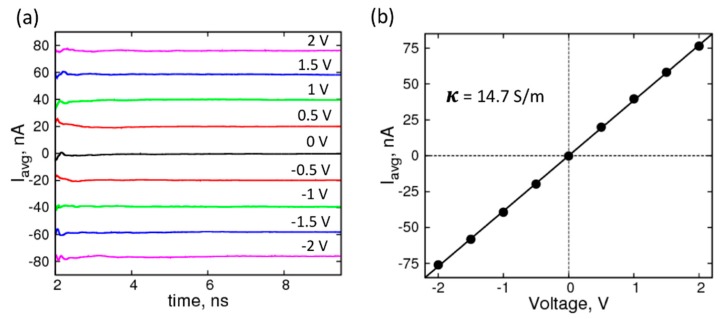
Characterization of the electrolyte (1 M KCl): (**a**) Average current profiles of simulation system with no graphene under forward and reverse biases of ±2 V (magenta), ±1.5 V (blue), ±1 V (green), ±0.5 V (red), 0 V (black); (**b**) I-V curve for the corresponding system with a straight-line fit yielding a conductivity (κ) of 14.7 S/m.

Our goal is to use MD simulations to predict changes in ionic current through the graphene nanopore due to the presence of RNA nucleosides. We began by characterizing the conductivity of the KCl solution at various concentrations using our simulation models and compared it to the experimentally measured conductivity of the electrolyte. We performed simulations at a range of applied constant voltages in the z-direction (from −2 V to +2 V) and calculated the response current by tracking the position of the ions, as outlined and validated by Aksimentiev and Schulten [46]. Briefly, the ionic current is calculated as a summation of the translation of the positive and the negative ions along the direction of the applied voltage, as given by the following equation:

(1) $$I\left(t\right)=\frac{1}{∆t\;L_{z}}\overset{n}{\underset{i=1}{\sum }}q_{i}\;\left\{z\left(t\right)-z\left(t-∆t\right)\right\}$$

where, *n* is the total number of K^+^ and Cl^−^ ions, *L*_z_ is the box-length in the direction (*Z*) of the applied voltage, *q_i_* is the columbic charge, and *z*(*t*) is the z-coordinate of the ions at time, *t*. The running averages of the currents, shown in Figure 2a, plateau well within the simulation timescale, indicating equilibration; and are therefore expected to remain unchanged if the simulations were extended. The average currents (Figure 2b) plotted against voltages yields an I–V curve that can be fit to a straight line to obtain resistance and conductance values. The molar conductivity of 1.0 M KCl solution for our system was 14.7 mS·m^2^· mol^−1^, which is 50% higher than the experimental value, is in good agreement with previous simulation studies, using different force-field parameter set and MD simulation package [47]. 

**Figure 3:**
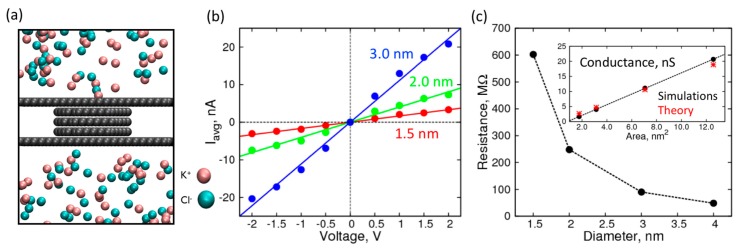
Characterization of empty nanopores of various sizes: (**a**) Simulation snapshot of the nanopore system; (**b**) I-V curves in 1 M KCl over a range of ±2 V through an empty nanopore made of 5-layered graphene of size 1.5 nm (red), 2.0 nm (green), and 3.0 nm (blue); (**c**) Resistance (MΩ) of the nanopore as a function of pore diameter. Inset: Comparison of theoretical (red asterisks), and simulated (black circles) conductance (nS) as a function of cross-sectional area of the pore.

Next, we characterized the conduction of ionic current through graphene nanopores (five atomic layers in thickness) of various diameters. We chose a five-layer nanopore because it can completely accommodate single nucleosides in our subsequent simulations, the results of which are discussed later. Like the control electrolyte simulations, we applied a constant voltage in the z-direction (perpendicular to the plane of graphene sheets) and obtained I-V curves for nanopores of the following diameters: 1.5, 2.0, and 3.0 nm (Figure 3b). The average current profiles are shown in Figure S1.

For voltages higher in magnitude than 2 V, we found that the I-V curves started to plateau and exhibited a sub-linear behavior, which can be attributed to the diffusion of the ions on the surface limiting the passage through the pore. Therefore, we used the linear range between −2 V to +2 V to calculate the resistance and conductance of the nanopores (Figure 3c). The resistance, when plotted against the pore diameter, decreases rapidly and asymptotically approaches zero. The resistance can be inverted to get conductance, which is shown in the inset, plotted against the cross-sectional area of the pore. As expected, the conductance is proportional to the cross-sectional area, showing a linear relationship with a near zero intercept. Furthermore, the conductance values are in good agreement with the expected theoretical values calculated by using specific conductance of 1.0 M KCl solution, using the formula below [48]:

(2) $$G=\frac{\pi \;d^{2}}{4h}\kappa$$

where *G* is conductance (Siemens), *d* is the diameter of the nanopore, *h* is the effective height of the nanopore, and *κ* is 14.7 S·m^−1^. The close correspondence between theory and simulations establishes a functional nanopore model system that can be used to localize biomolecules and observe changes in its conductance/resistance.

**Figure 4:**
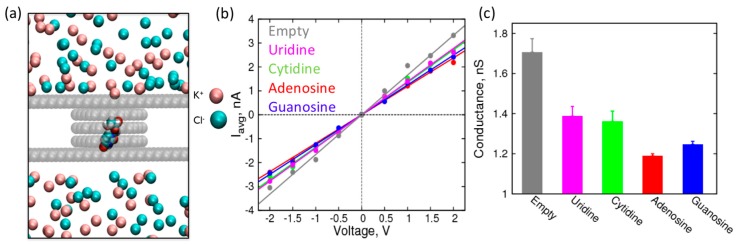
Characterization of a 1.5 nm graphene nanopore in the presence of canonical nucleosides: (**a**) Simulation snapshot of the system showing nucleoside localization to a nanopore. Van der Waals representation is used for the graphene carbon atoms, nucleoside, and ions. Water molecules are excluded for clarity; (**b**) I-V curves with their corresponding straight line fits for empty 1.5 nm pore (grey), pore with canonical uridine (magenta), cytidine (green), adenosine (red), and guanosine (blue) localized at the center of the nanopore in 1 M KCl; (**c**) Resistance (MΩ) of 1.5 nm pore with canonical nucleosides.

Following the characterization of the conductance of the nanopore over a range of pore sizes, we introduced RNA nucleosides in the nanopore to observe their effect on the resistance of the pore. In the current study, we localized the nucleosides to the pore using harmonic constraints in *z*-direction at the center of the pore (Figure 4a). To enable the nucleoside to freely adopt its preferred conformations consistent with its interactions with the walls of the nanopore, we allowed freedom of movement in the *x*- and *y*-direction. 

The conformation adopted by the nucleosides in the heterogeneous environment of the nanopore is going to be highly dependent on the models used for nucleoside–graphene interactions. The interaction of individual nucleosides to graphene was shown to be highly over stabilized using the default AMBER parameters [49]. Therefore, we used the newly developed AMBER-type force field parameters for nucleoside–graphene interactions, which employed gas phase quantum mechanical calculations, and calibrated against experimental thermodynamic results [49]. The calibrated force field was shown to reproduce the thermodynamic behavior of nucleosides and oligonucleotides at the graphene surface, an important parameter for characterizing the structure and interactions of nucleic acids at graphene–water interfaces. 

First, we performed simulations of canonical nucleosides and calculated the conductance of the pore when the nucleosides are localized to it (Figure 4b,c). The conductance values are derived from average currents shown in Figure S2. While all the nucleosides decrease the conductance of the nanopore by blocking the passage of ions, it is interesting to note that the purines have a distinctly higher increase in resistance compared to the pyrimidines. This observation is consistent with the size of the nucleosides: the purines have larger ring structures, which expectedly have a larger steric effect compared to the pyrimidines [2,50]. Furthermore, among the purines (or pyrimidines), the differences in the conductance values are significant (outside the error bars) to resolve their identity (adenosine vs. guanosine and uridine vs. cytidine). This difference in conductance (or ionic current) is already being exploited for nanopore-based sequencing of DNA oligonucleotides (MinION, Oxford Nanopores Technologies, Oxford, UK). However, to accurately predict the change in ionic current while an oligonucleotide is translocating through the pore, we need more sophisticated simulations of oligonucleotides. Nevertheless, this study on localized nucleosides provides a good starting point to assess the feasibility of detection of nucleosides based on changes in ionic current or pore conductance. 

While previous results suggest possible differentiation of canonical nucleosides using nanopores, the capability of the nanopore to distinguish modified RNA nucleosides from their canonical counterparts is unknown. tRNAs are known to be the most modified RNA, in particular having modified uridines in the anti-codon stem-loop that enable them to decode cognate and near-cognate codons at the wobble position [51]. Uridine modifications have been well studied, some of which (cm^5^U, mcm^5^U, mcm^5^Um) play a role in the synthesis of selenoproteins essential for oxidative stress response in cells. The focus of our research is on uridine modifications which include: cm^5^U, mcm^5^U, mcm^5^Um, and mcm^5^s^2^U.

**Figure 5:**
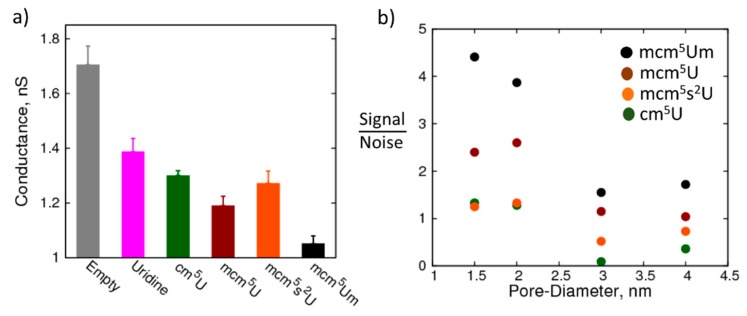
Detection of modified nucleosides using the nanopore: (**a**) Resistance of a 1.5 nm pore with canonical uridine and modified uridine immobilized at the center of the pore: empty pore—grey, uridine—magenta, 5-carbonyl-methyluridine (cm^5^U)—dark green, 5-methoxycarbonyl-methyluridine (mcm^5^U)—maroon, 5-methylcarboxymethyl-2-thiouridine (mcm^5^s^2^U)—orange, and 5-methoxycarbonyl-2′-*O*-methyluridine (mcm^5^Um)—black; (**b**) Degree of sensitivity (gamma, expressed as percentage difference in resistance between uridine and modified uridine, relative to uridine) decreases as pore diameter increases.

Figure 5a shows the conductance of a 1.5 nm pore when canonical and modified uridine nucleosides are localized in the nanopore (please refer to Figure S3 for the average current profiles). Remarkably, the change in conductance of the nanopore is significant even for the smallest modification that we studied (cm^5^U). Further, with increase in the size of the modification the resistance of the nanopore progressively increases, which suggests that the nanopore resistance is sensitive to these changes. However, with mcm^5^s^2^U in the pore, resistance decreases compared to mcm^5^U. This decrease is likely due to the enhanced Lennard-Jones (LJ) interactions of sulfur with the walls of the nanopore without significant increase in the size of the molecule, which is part of our ongoing investigation. Importantly, the pore resistances are distinct for the modifications allowing for differentiation between the modifications. Overall, the 1.5 nm pore serves well to detect the changes in the modification chemistry. 

How does the detection capability change when the size of the nanopore is increased? Can larger nanopores (which are easier to fabricate) still detect the modifications? To that end, we studied the resistance of nanopores with larger diameters in the presence of the canonical and modified uridine nucleosides. Figure 5b shows the signal to noise ratio of the conductance of the pore between uridine and its modifications, cm^5^U, mcm^5^U, mcm^5^Um, and mcm^5^s^2^U, highlighting the change in ability of the nanopore to detect the modification with increase in the pore diameter. The signal is the difference in the conductance of the nanopore between the canonical and the modified nucleoside, while the noise is the sum of standard deviations in the conductance obtained from block averaging for the corresponding nucleosides. While the largest modification studied here (mcm^5^Um) was still distinguishable from the canonical nucleoside at even a 4.0 nm pore size, the smaller modifications have a sub-one signal to noise ratio at pore radii greater than 2.0 nm. It is also interesting to note that the change in conductance between mcm^5^U and mcm^5^Um is higher than that between U and cm^5^U, even though the size of the functional group is smaller in the former compared to the latter. This observation implies that in addition to the size of the functional group, the location of the modifications and the chemistry also play an important role in modulating the passage of ions through the nanopore. Therefore, we can conclude that nanopores have the potential to distinguish between small modifications and differentiate between isomers, such as methylations at different positions of the nucleoside, which is part of the ongoing work in our labs.

## 3. Materials and Methods 

We performed three sets of MD simulations: (A) blank electrolyte, (B) empty nanopore, and (C) nucleosides in nanopore. While the simulation setup is different for the three different sets, the methodology and run parameters were the same. The simulation box dimensions were 5.16 × 5.10 × 10.0 nm^3^ for all the three simulation sets. The blank electrolyte simulations (A) of 1 M KCl contained 8400 water molecules, and 160 K^+^ and Cl^−^ ions. For the nanopore simulations (B and C), Visual Molecular Dynamics (VMD 1.9.2 version) [52] software was used to generate a five-layered graphene of arm chair edge type with C–C bond length of 0.1418 nm, and sheet dimensions of 5.16 nm × 5.10 nm. A simulation box of above-mentioned dimensions was then created with a five-layered graphene sheet with a nanopore at the center. In the five-layered graphene, we removed the carbon atoms that were within “d” diameter from the center of the sheets to create a nanopore with atomic smoothness. For improving computational efficiency, we removed the carbon atoms in the middle sheets that are not directly exposed to the inside of the nanopore. All the carbon atoms of the graphene sheet are kept frozen throughout the simulation. While water molecules (~6400) and ions (~130 K^+^ and Cl^−^) were added to system, ensuring no overlap of atoms for the simulations in set B, a canonical or modified RNA nucleoside is carefully placed at the center of the nanopore before addition of water molecules and ions for simulations in set C. The nucleosides were immobilized in the center of the graphene nanopore by using a harmonic potential in the z-direction, with complete freedom of movement in the *x*- and *y*-directions. Nucleoside modifications were achieved by using the WebMO graphic editor (WebMO Enterprise, WebMO LLC, Holland, MI, USA) to add cm^5^U, mcm^5^U, mcm^5^s^2^U, and mcm^5^Um modifications to canonical U. Force fields and partial charges on the nucleoside atoms were generated using q4md force-field tools from the RESP ESP charge Derive Server (REDS) [53]. Partial charges were derived using Hartree-Fock level theory and 6-31G* basis-sets with energy minimization of the geometry of the nucleosides generated. We used AMBER-99 type force-field parameters for bonded interactions. LJ parameters employing Chen-Garcia corrections were used for the nucleosides [54]. LJ corrections for the ion parameters are obtained from Chen and Pappu, to ensure no aggregation of monovalent ions at molar concentrations [55]. For nucleoside-graphene interactions, LJ parameters from the recent calibration work were employed. Graphene parameters were chosen from Werder et al. [56] that were parameterized against contact angles of micro droplets of water on graphene surface. For nucleotide carrying a net charge, the system was made charge-neutral by adjusting the number of potassium and chloride ions. 

All simulations were performed using the Gromacs-4.6.3 package [57]. Integration of equations of motion and propagation of trajectory were done using Leapfrog algorithm with a 2 ps time step. Temperature of the system was maintained at 300 K using the V-rescale thermostat [58]. Semi-isotropic pressure coupling was achieved using the Berendsen algorithm [59] at 1 atmospheric pressure to establish an NPT ensemble. The Particle Mesh Ewald algorithm with a real space cutoff of 1.3 nm was used to calculate the long-ranged electrostatic interactions. LJ interactions were also cut off at 1.3 nm. Water molecules were represented using the TIP3P model [60]. The motion of hydrogen atoms bonded to heavy atoms was constrained using the LINCS constraint algorithm of order 4. The graphene sheets were frozen in the *xyz*-direction, and the electric field was applied only in the *z*-direction. The blank electrolyte and empty pore simulations were run for 10 ns that included 2 ns of equilibration and 8 ns of production time, while the nucleosides simulations were run for 50 ns, of which 10 ns were used to allow the system to equilibrate. Coordinates of ions were stored every picosecond for calculation of ionic current. The equilibration part of the simulation is not included in the calculation of the current, and the average current profiles were produced using running averages of the instantaneous current. Furthermore, the production part of the simulation is divided into five blocks, and block averages were used to estimate the error bars. This work used the Extreme Science and Engineering Discovery Environment (XSEDE) [61].

## 4. Conclusions

Graphene nanopores have been widely studied and are currently in use for DNA sequencing and similar applications. Solid-state devices made of graphene have superior electrical and mechanical properties, which hold promise for next-generation devices for detection of biomolecules. The heightened interest in the field of epitranscriptomics that deals with post-transcriptional modifications of RNA has sparked an interest in detection and quantifications of RNA modifications, especially the ones in tRNAs. The potential of graphene nanopores for the detection of RNA modifications has not yet been explored. To that end, we have here presented the possibility of tRNA modifications detection in graphene nanopores using molecular dynamics simulations. First, we showed that our MD simulations and ionic current characterization are consistent with previous simulation studies. Secondly, by using experimentally calibrated parameters for RNA–graphene interactions, we characterized the nanopores resistance in the presence of canonical and modified nucleosides, and showed increase in the detection capability of the nanopore by reducing the nanopore dimension. Thirdly, we showed that the ionic current through the nanopore is sensitive to the size of the modification chemistry and is also dependent on the functional group of the modification and its location on the nucleoside. These findings suggest that carefully constructed nanopore devices can potentially be used for detection of a wide range of RNA modifications, including the ones that are isomeric to each other. Our studies provide a good foundation for the design, fabrication, and optimization of a device for bedside applications to facilitate biomarker analyses for potential disease diagnostics, and characterize the epitranscriptome. Our simulation methodology includes unique molecular models or force-fields that were specifically calibrated and validated against experimental observables, including thermodynamic behavior of nucleotides in solution and bound to graphene. Our future studies will also incorporate improved models for ions that are part of ongoing work. This approach is applicable to other simulations studies where the models are tested against experiments before being applied to draw new insights and conclusion. While the current study focused on specific tRNA modifications found on wobble uridines, future studies include modifications of other nucleosides and fabrication and characterization of nanopores that are informed by our current findings.

## Supplementary Materials

The following are available online at www.mdpi.com/2218-273X/7/3/65/s1, Figure S1: Average current profiles of empty pores derived from 10 ns trajectories, Figure S2: Average current profiles of single canonical nucleosides derived from 40 ns trajectories in 1.5 nm pore, Figure S3: Average current profiles of single modified and unmodified uridines derived from 40 ns trajectories in 1.5 nm pore.
