# Supplementary material for: tRNA Modification Detection Using Graphene Nanopores: A Simulation Study
Source: Biomolecules. 2017 Aug 25;7(3):65. doi: 10.3390/biom7030065 (PMC5618246; doi:10.3390/biom7030065)
Supplement: Supplementary file 1 [file biomolecules-07-00065-s001.pdf]

SUPPLEMENTARY FIGURES.

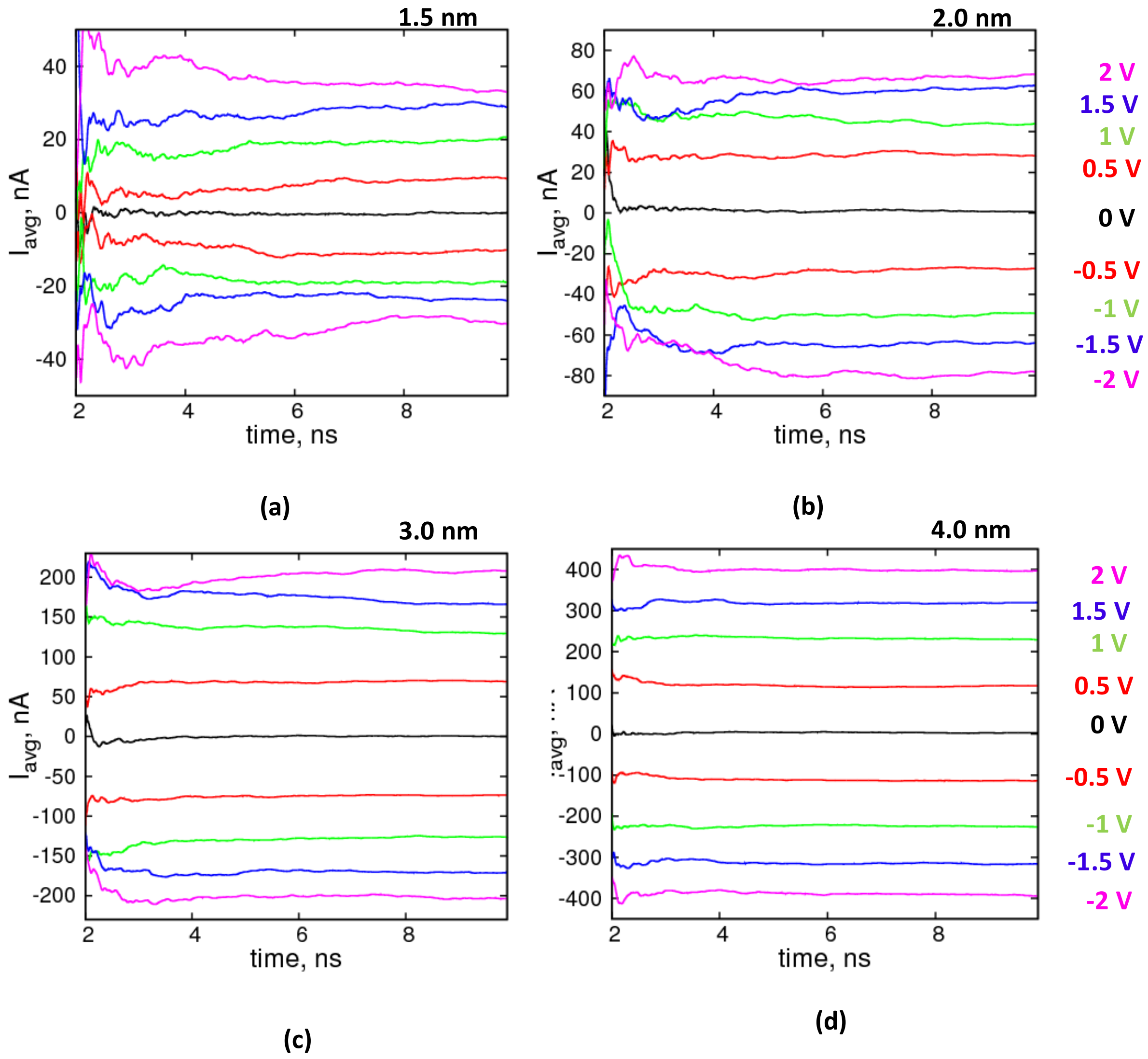

Figure S1. Average current profiles of empty pores derived from 10 ns trajectories in (a) 1.5 nm (b) 2.0 nm (c) 3.0 nm (d) 4.0 nm pore with 1.0M KCl.

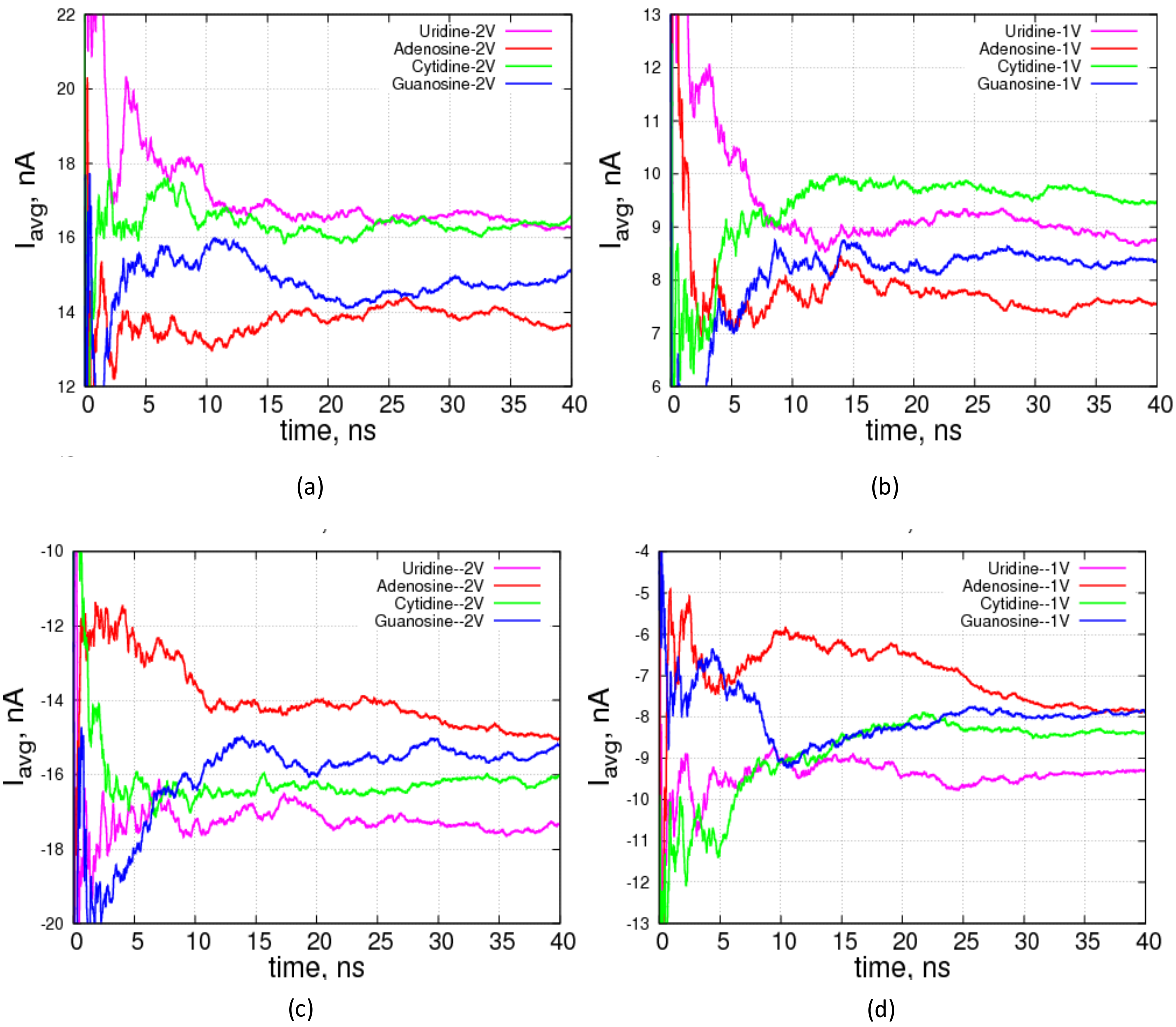

Figure S2. Average current profiles of single canonical nucleosides derived from 40 ns trajectories in 1.5 nm pore with 1.0 M KCl and (A) +2V, (B) +1V, (C) -2V, (D) -1V; Uridine- magenta, Adenosine-red, Cytidine-green, and Guanosine- blue.

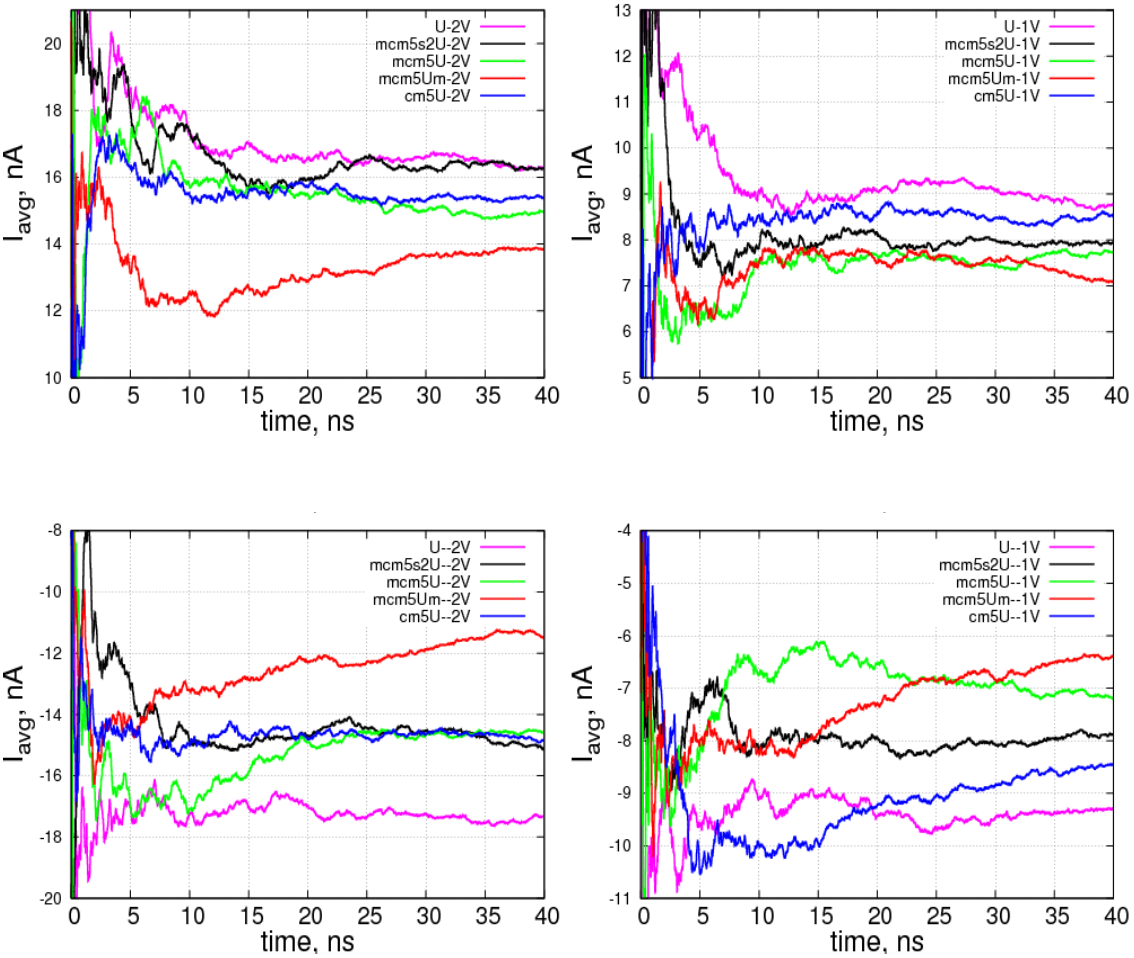

Figure S3. Average current profiles of single modified and unmodified uridines derived from 40 ns trajectories in 1.5 nm pore with 1.0M KCl and (A) +2V, (B) + 1V, (C) -2V, (D) -1V; Uridine- magenta, 5-carbonyl-methyluridine (cm^5U)-blue, 5-methoxycarbonyl-methyluridine (mcm^5U)- green, 5-methoxycarbonyl-2′-O-methyluridine (mcm^5Um) – red, and 5-methylcarboxymethyl-2-thiouridine (mcm^5s^2U)- black.
